# Reinforcements and augmentations with the long head of the biceps tendon in shoulder surgery: a narrative review

**DOI:** 10.1530/EOR-2024-0122

**Published:** 2025-05-05

**Authors:** Alberto Guevara-Alvarez, Edwin Valencia-Ramón, Alejandro Lopez-Villers, Luis Fernando Navarro-Pérez, Israel Gonzalez-Rizo, Gonzalo Eduardo Gomez, Alexandre Laedermann

**Affiliations:** ^1^ Instituto de Hombro IDH, Hospital Angeles Centro Sur, Querétaro, Mexico; ^2^ Instituto Queretano de Alta Especialidad en Ortopedia IQAEO. Hospital Angeles Centro Sur, Querétaro, Mexico; ^3^ Hospital Sportsmed Global, Buenos Aires, Argentina; ^4^ Division of Orthopaedics and Trauma Surgery, La Tour Hospital, Meyrin, Switzerland; ^5^ Faculty of Medicine, University of Geneva, Geneva, Switzerland; ^6^ Division of Orthopaedics and Trauma Surgery, Department of Surgery, Geneva University Hospitals, Geneva, Switzerland; ^7^ FORE (Foundation for Research and Teaching in Orthopedics, Sports Medicine, Trauma, and Imaging in the Musculoskeletal System), Meyrin, Switzerland

**Keywords:** glenohumeral instability, superior capsular reconstruction (SCR), rotator cuff repair, anterior cable reconstruction (ACR), shoulder instability, rotator cuff augmentation (RCA), dynamic anterior stabilization (DAS)

## Abstract

- The long head of the biceps tendon (LHBT) has recently emerged as a therapeutic option for various shoulder pathologies.
- Synthetic materials and allografts have not shown sufficient resistance or favorable outcomes to restore rotator cuff native tissue properties, leading to consideration of using LHBT as biological augmentation.
- LHBT mimics adjacent structures, such as the rotator cuff, is easily accessible during surgery, and is a good source of live autologous cells for regenerative augmentation in rotator cuff repair, as a superior capsular reconstruction in irreparable cuff tears, in subscapularis augmentation in shoulder replacement and as a stabilizer in anterior shoulder instability.
- This narrative review aims to collect, synthesize and critically evaluate the literature on the use of the LHBT and its current applications in the field of shoulder surgery, improving the understanding of the terminology and consolidating the concepts related to the various procedures in shoulder surgery.

## Introduction

The long head of the biceps tendon (LHBT) is known to cause shoulder pain and tenotomy or tenodesis are widely used successful treatment techniques, particularly when combined with rotator cuff injuries (1, 2).

As surgical techniques advance, synthetic materials and allografts have not shown sufficient resistance or favorable outcomes to restore rotator cuff native tissue properties, leading some to consider using LHBT as biological augmentation (3), since it mimics adjacent structures like the rotator cuff. It is easily accessible during surgery (4) and is a good source of live autologous cells for regenerative augmentation (5).

LHBT has proven being useful for surgical reconstruction, including as an autograft during superior capsular reconstruction (SCR) for rotator cuff tears (4, 6), as an augmentation of poor soft tissue quality during rotator cuff repair (RCR) or shoulder arthroplasties (7) and even to reinforce the capsule (8) or reconstruct a missing labrum for glenohumeral stabilization (9).

This narrative review therefore aims to collect, synthesize and critically evaluate the literature on the use of the LHBT and its current applications in the field of shoulder surgery.

## Methods

### Literature search strategy

We conducted a narrative review of the literature regarding the use of LHBT and its current applications in shoulder surgery to filter the most suitable articles. We searched in PubMed.gov (http://www.ncbi.nlm.nih.gov/pubmed) using MeSh terms, the use of the LHBT in RCR as (‘irreparable rotator cuff tears’ OR ‘irreparable rotator cuff tear’ OR ‘massive rotator cuff tears’ OR ‘massive rotator cuff tear’ OR ‘RCR’ OR ‘rotator cuff augmentation (RCA)’ OR ‘anterior cable reconstruction (ACR)’ or ‘superior capsular reconstruction’ OR ‘anterior shoulder dislocation’ OR ‘anterior shoulder stabilization’ OR ‘subcritical bone loss treatment’ OR ‘subcritical glenoid bone loss’ OR ‘dynamic shoulder stabilization’ OR ‘dynamic anterior stabilization (DAS)’ OR ‘anterior shoulder stabilization’) AND (‘long head of biceps’ OR ‘biceps long head’ OR ‘LHBT’ OR ‘biceps’).

### Inclusion criteria

The inclusion criteria were established as follows:

- Articles published in English and Spanish.
- Articles published in a peer-reviewed journal.
- Prospective or retrospective clinical studies.
- Between 2017–February 2023.
- Adult population diagnosed with rotator cuff tear or shoulder instability.
- No racial or gender limits.
- Intervention: surgical treatment, whether it has a postsurgical intervention of RCR, RCA, augmentation of subscapularis repair, SCR or shoulder instability with LHBT.
- In our narrative review, we decided to include studies describing different surgical techniques with the use of LHBT in various shoulder pathologies. Prospective and retrospective clinical studies to observe the results of the various techniques. Biomechanical studies to know the potential favorable or adverse effects of the use of LHBT.

### Exclusion criteria

Exclusion criteria were letters to the editor, case reports, reviews, commentaries, studies conducted on animals and papers that did not include the purpose of the work, tenodesis vs tenotomy papers, use of LHBT for a different purpose rather than RCR, SCR, ACR and shoulder instability.

### Study selection and data extraction

Retrospective or prospective studies, technical notes, clinical and biomechanical studies, randomized-controlled trials or nonrandomized controlled trials, in which the uses of LHBT in different procedures, such as RCR, SCR, augmentation of subscapular repair and shoulder instability were analyzed. One author (LFN) independently selected studies with inclusion criteria by screening titles and abstracts. The author reviewed the full texts of the selected studies to determine the inclusion articles. Disagreements were resolved in consultation with the lead author.

### Quality assessment

For clinical studies, two reviewers (AG and LFN) critically appraised the studies for potential sources of bias. For studies describing surgical techniques, the adequacy of the description of the procedure details, any adjuvant procedures and the postoperative rehabilitation was evaluated.

## Results

### Search results

**Figure 1:**
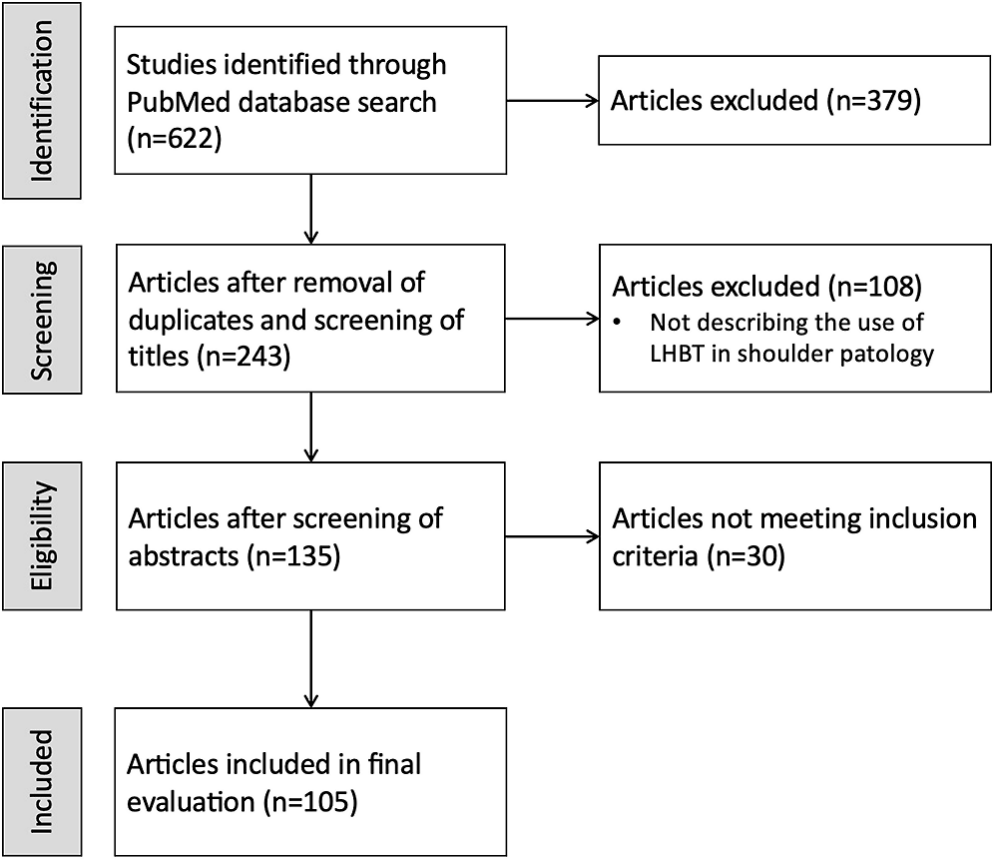
Article search flow diagram for narrative review.

We found 622 citations in the initial literature search. After removing duplicates, we were left with 243 articles. 108 articles were excluded due to irrelevance and 30 more were excluded after additional screening. We selected 105 articles for review: 14 biomechanical studies and 91 clinical studies, including 35 surgical techniques (Fig. 1).

### Surgical techniques studies

We found 16 technical notes using LHBT for irreparable rotator cuff tears, four techniques using LHBT for ACR, 11 techniques for glenohumeral instability with subcritical glenoid bone loss and four techniques for subscapularis repair following shoulder arthroplasty.

### Clinical studies characteristics

We analyzed 86 clinical studies from around the world. They covered the different uses and techniques of LHBT. LHBT was used in SCR and RCA in 60 studies, in ACR in 11, in shoulder instability in 11 and augmentation of subscapularis repair in four studies.

### Biomechanical studies characteristics

14 biomechanical studies were analyzed, including two on LHBT techniques for repair, reconstruction and augmentation. Four studies were conducted in Asia, one in Europe and nine in North America. The studies covered different uses and techniques of LHBT, including its use in SCR, RCA and ACR.

## Discussion

### Rotator cuff tears

#### SCR

##### Introduction

Treatment of massive rotator cuff tears is challenging due to several factors including muscle infiltration, tendon retraction and tissue degeneration. Despite surgical advancements, rerupture rates remain high, up to 94% (10, 11, 12, 13, 14, 15).

The Bush procedure (16) was developed in 1975 as a treatment for massive injuries in patients with poliomyelitis-induced paraplegia. It prevented humeral head escape by repositioning the LHBT more laterally (17, 18). In 2001, Guven *et al.* (19) introduced the concept of SCR with the LHBT, which increased the tendon’s anteroposterior diameter by making an incision between the tendons. Their study of 14 patients diagnosed with an irreparable supraspinatus injury showed positive results on the Constant score (20) and patient satisfaction.

Different materials have been used for SCR, such as xenografts (21, 22, 23, 24) and dermal (25, 26, 27, 28, 29) allografts. While these materials have improved patient functionality in Constant and ASES scores (27, 30), complications and retear rates of up to 20–70% (22, 24, 30, 31) remain an issue, and commercially available scaffolds are expensive (32). One alternative is using LHBT (33) as a potential autograft for rotator cuff tears. LHBT (see Supplementary Material 1 (see section on Supplementary materials given at the end of the article)) can provide a free, easily accessible and direct option for RCA, without concerns of immune reaction or architectural misalignment (32, 34).

##### Biomechanics

Mihata *et al.* in 2012 (35) formalized SCR as a part of treating irreparable rotator cuff injuries. SCR limits superior migration of the humeral head, increases subacromial space and improves the capsular depressor effect and lever arm, leading to better clinical outcomes.

Some studies have explored the LHBT’s potential as an injector in RCA (36). Denard *et al.* (37) introduced the ‘bicep box’ technique, where the LHBT is secured anterolaterally, posterolaterally and posteromedially. The technique may help augment irreparable lesions (37, 38) by restoring superior translation and peak subacromial contact pressure (29).

Berthold *et al.* investigated three techniques described for SCR using the LHBT and reported that shoulder function is improved by decreasing glenohumeral superior translation, maximum cumulative deltoid force and subacromial pressure contact point (6).

LHBT is a stronger autograft option for SCR than fascia lata, according to El-Shaar *et al.*’s (39) study. The study found that glenohumeral superior translation occurred at almost 400% of the force required in the context of a massive tear with LHBT, while it occurred at only 194% with fascia lata. Chen *et al.* (40) used the LHBT as an autograft for superior labral reconstruction. This prevents superior glenohumeral escape and arthrosis in cases of irreparable rotator cuff lesions. The study showed two techniques that restore superior glenohumeral restraint.

##### Recent techniques

**Figure 2:**
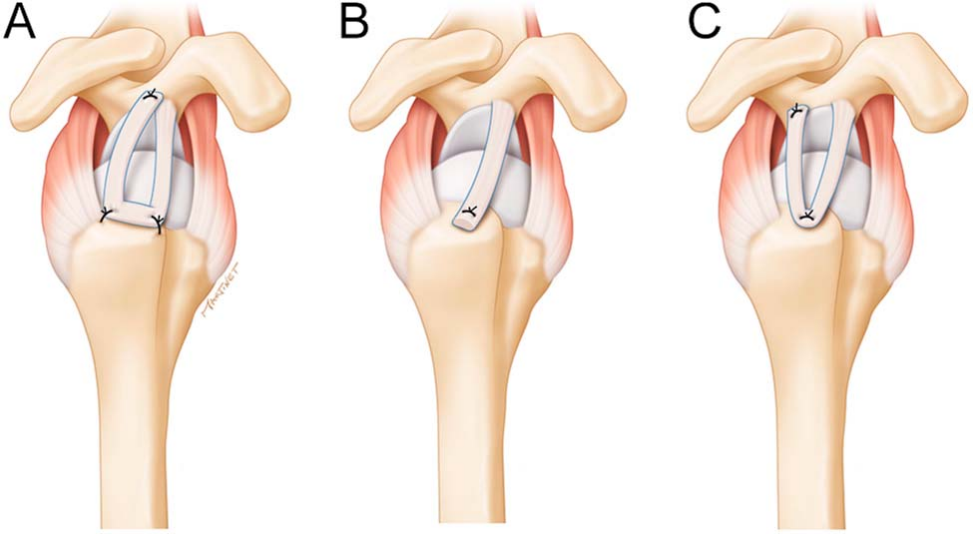
Three types of configurations for SCR with autologous LHBT. The common aspect of these techniques is the preservation of the glenoid origin of the LHBT, (A) box shape technique with two anchors at the footprint of supraspinatus and one anchor at the superior glenoid area to fix the loose end of the LHBT, (B) anterior cable reconstruction with a single anchor at the anterior portion of the footprint area, (C) U-shape technique with a single medial anchor at the humerus and the loose end of the tendon brought back into the glenoid to be fixed with one more anchor. SCR, superior capsular reconstruction; LHBT, long head of the biceps tendon. Reproduced with permission from https://beemed.com/.

In 2017, Boutsiadis (41) *et al.* developed a new technique using the LHBT for SCR. This technique resulted in good clinical outcomes (37, 42) and fewer complications compared to the use of allograft (42). Different surgical techniques using only the intra-articular portion of the LHBT or both the intra- and extra-articular portions have since been developed. The common aspect of these techniques is the preservation of the glenoid origin of the LHBT, which contributes to positive outcomes and decreases the rate of rerupture. Another variation involves performing an SCR with the LHBT in its entirety (Fig. 2).

There are different techniques for SCR. Some techniques preserve the glenoid origin of the LHBT (34, 41, 43, 44, 45, 46), such as those proposed by Barth *et al.* (47) and Kim *et al.* (44, 45). This approach allows for partial RCR and avoids potential pain. Preserving the LHBT’s glenoid origin decreases rerupture rates and maintains stability (47). Another variation involves positioning the LHBT anterior (48) or posterior (46) to the supraspinatus, depending on the rotator cuff lesion morphology (49). Some works (50) have used the intra and extra-articular portion of the LHBT.

The extra-articular portion of the LHBT is used to create a double bundle of LHBT for the reconstruction of the superior capsule. The technique triples the length of the graft, and a subpectoral tenodesis of the remnant of the LHBT is performed in the form of an ‘S’ (50) or snake. This approach has shown improvement in functional scores and obtains an integrity of the repair up to 86.7% of cases in MRI (50). The LHBT is first attached to the greater tuberosity and finally to the glenoid, giving a ‘U’ shape (38). The technique involves passing the extra-articular portion of the LHBT through an anteroposterior bone tunnel in the humeral head and finally joining it to the posterosuperior glenoid segment in the form of a transosseous loop (51).

Brandao *et al.* (51) reported the ‘biceps loop technique’ for young and active patients with irreparable rotator cuff tears. The technique involves tenodesis of LHBT, releasing the extra-articular portion of LHBT, drilling a bone tunnel, moving the LHBT posteriorly and fixing it to the posterosuperior glenoid rim. The technique recreates joint fulcrum and prevents progression to cuff tear arthropathy (51).

##### Arthroscopic techniques without LHBT tenotomy

In 2020, Kim *et al.* (45) reported a technique for using the LHBT without undergoing tenotomy. This preservation approach involves an L-shaped detour to maintain LHBT tension and fixing the LHBT to the greater tuberosity center. Another technique involves a new bicipital groove and releasing the transverse ligament to alter the entire LHBT route (52).

#### Augmentation of RCR

##### Introduction

**Figure 3:**
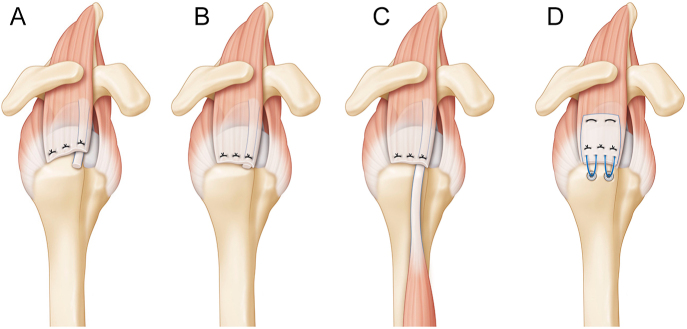
Different options for augmentation in rotator cuff repairs. The LHBT can be used to create a mechanically expanded autograft or scaffold to augment the RCR; in these figures we summarized them (A) partial repair with LHBT augmentation and tenotomy, (B) full repair with LHBT augmentation and tenotomy, (C) full repair with LHBT with biceps rerouting into the greater tuberosity without tenotomy and (D) as a compressed biceps patch to enhance the rotator cuff tendon repair. (LHBT, long head of the biceps tendon; RCR, rotator cuff repair). Reproduced with permission from https://beemed.com/.

The LHBT can be used to create a mechanically expanded autograft or scaffold to augment the RCR (53, 54). It has been used in various ways, including as a free graft (55), pediculated autograft (56) and to reinforce the repair of massive tears of the anterior rotator cuff (57). Techniques involve rerouting, tenodesis and suturing to improve healing potential and decrease tension on the repair (31). Chiang *et al.* (43, 58, 59), Cañete (60), Memon (61) and Ballesteros *et al.* (52) have used the LHBT as a reinforcement for RCR. The technique involves placing the LHBT between two rotator tendons. Bhatia used the LHBT and the subacromial bursa to augment RCR. They performed a tenodesis of the LHBT using subscapularis repair sutures and used its proximal stump as a graft for ACR, along with the bursa (62) (Fig. 3).

##### Laboratory studies

Colbath *et al.* (32) described LHBT as a potential scaffold for biological cuff augmentation using a surgical graft expander. The scaffold can be altered or crushed to produce bioactive signals, supporting cuff repair augmentation. LHBT can be molded into a patch or used as a crushed graft, depending on the rotator cuff’s repairability, tear severity and tendinopathy level (32).

##### Patch augmentation

Selim *et al.* described a technique for augmenting a massive rotator cuff tear with LHBT by folding the tendon back on itself (63). Tokish *et al.* and Endell *et al.* reported using the LHBT in a patch to enhance cuff tendon repair (5, 64, 65).

##### Clinical results

Procedures involving the LHBT may decrease structural failures compared to isolated repairs or allogenic dermal patches, although their clinical benefit remains unclear (66).

In a study comparing arthroscopic repairs of massive rotator cuff tears with or without LHBT augmentation, significant differences were found. The use of LHBT augmentation showed twice the rate of repair healing on magnetic resonance imaging (67). The technique involved using the LHBT as an internal splint to increase tendon–bone contact and decrease tension on the repaired site. Similar results were reported in another study comparing repairs with and without rerouting of the LHBT (66).

Lin *et al.* (68) reported three patients with full-thickness rotator cuff tears who were treated with a double-row technique and autograft augmentation. At the 6-month follow-up, the VAS score decreased from 6.33 to 1.23, and the Constant score increased from 45.33 to 88 (68).

In a study of 77 patients, Park *et al.* found no significant difference in retear rates between partial arthroscopic repairs with and without incorporating the LHBT, despite a retear rate decreased by half with LHBT incorporation (69). Llinás *et al.* compared outcomes in patients with massive rotator cuff tears who underwent repair alone (*n* = 50) or repair with partial SCR (*n* = 56). A 2-year follow-up with ultrasound showed significantly lower rerupture rates and better clinical scores in the group that had partial SCR repair and LHBT (70).

#### ACR

##### Introduction

In massive rotator cuff tears, repairing only tendinous structures without addressing the anterior capsule may result in higher retear rates for tears of the anterior supraspinatus portion involving the anterior cable compared to semilunar tears (71, 72). The superior capsule thickens into a cable that acts as a passive stabilizer, while the tendinous cord from the supraspinatus tendon serves as a dynamic stabilizer, maintaining the humeral head within the joint and preventing superior migration (Table 1) (71, 72, 73, 74).

**Table 1:** Summary of research papers of ACR.

Study	Country	IND/CONTRA	Technique	Results	Conclusions
Shin *et al.* (74)	Korea	IND: For anterior L-shaped RCT	• ACR using the biceps • Debride adhered tissue and adhesive capsule around the retracted supraspinatus • Insertion of a medial anchor • Tenodesis of the proximal portion of LHBT with two strands wrapping around the biceps • Tenotomy and tenodese distal portion of biceps • Lateral anchor for reduce and fix of tendon and fixation • Suture the supraspinatus and infraspinatus to LHBT.	It is possible to repair retracted supraspinatus with the LHBT tenotomized, minimizing exposed footprint which could reduce tension and increase healing rate. Imbalance couple forces of rotator cuff may occur by anterior mobilization of the infraspinatus	ACR using LHBT is a surgical option for patients with retracted anterior L-shaped RCT. The tenodesed proximal biceps tendon can act as the anterior part of the rotator cable and reinforce RCR by suturing the torn cuff together
		The biceps tendon must be intact			
Chen *et al.* (75)	USA	IND: For repairable large-to-massive RCT	• ACR with LHBT • Subacromial bursectomy • Debride bone bed at greater tuberosity • Tenodesis of LHBT at superior aspect of the bicipital groove • Insert anchor superior to the biceps groove • Suture through LHBT and through anterior rotator interval tissue • Release the proximal biceps origin	LHBT reconstruct and augment the anterior rotator cable, leaving the tendon in continuity on the humeral side. LHBT is attached to the glenoid and serve as a scaffold for reduction of the rotator cuff	This technique could improve tissue quality of the repaired construct and enhance repair longevity
		The biceps tendon must be intact			
Giacomo *et al.* (76)	USA	IND: Large-to-massive RC defects	• ACR using proximal LHBT • Infraspinatus tendon repair • Greater tuberosity preparation • Attach graft to capsular footprint • Repair native posterosuperior capsule to biceps tendon • Fix biceps tendon with a lateral anchor	This ACR restores the repaired dynamic tendon and reactivates the underlying capsule to improve function and glenohumeral congruency. Bone troughs increase healing biology	Lower cost and avoids another interface for possible failure by not using anchors. Use of LHBT and its tenotomy may cause pain and esthetic deformity
		Biceps must be intact			
		Infraspinatus or supraspinatus tendon should be repairable			
Llanos- rodriguez *et al.* (48)	Spain	IND: Massive rotator cuff tears, Anterior L-Shaped RCT	• Debridement of soft tissue around the LHBT • Create a trough for rerouting the LHBT • Anchor insertion in the trough to fix the LHBT • Use two anchors with two strands • One lasso loop suture • One suture over the tendon • Transfer and tenodesis of LHBT • RCR		Prospective studies with large cohort populations and long-term follow-up are necessary to establish the effectiveness of the technique


IND, Indication; CONTRA, contraindications; RC, rotator cuff; RCT, rotator cuff tear; RCR, rotator cuff repair; ACR, anterior cable reconstruction; LHBT, long head of the biceps tendon.

In partial RCRs without ACR, the repaired tendon bears all the forces. To enhance stability, the LHBT is transferred to the front edge of the repaired rotator cuff (Fig. 2).

##### Current techniques

Shin *et al.* (74) described a technique involving the repair of the retracted supraspinatus tendon at the proximal LHBT, emphasizing the importance of LHBT orientation to reduce tension during repair, with favorable functional outcomes and decreased rerupture rate (77). Chen *et al.* released the glenoid origin of the LHBT in their ACR technique, attaching the tenotomized portion of the LHBT to the footprint of the rotator cuff at the greater tuberosity, resulting in the loss of the LHBT’s mechanical property, rendering it solely a biological augment (75).

De Giacomo *et al.* (76) described a technique focusing on restoring anterior loading rather than covering the entire humeral head with the LHBT autograft. They believed that preparing the bony depression in the greater tuberosity increases the area of contact with the transferred tendon graft, facilitating healing biology and improving graft stability during shoulder rotational movements (76).

Llanos-Rodriguez *et al.* focused on using the LHBT as a static stabilizer to promote healing of posterosuperior rotator cuff tears. The LHBT was transposed along a new bone path, followed by tenotomy distal to its insertion point, and then the posterosuperior repair of the rotator cuff (48).

##### Clinical results

Barth *et al.* (47) studied 82 patients with massive cuff tears who underwent repair using different techniques. The study found that patients in the ACR with LHBT group had better tendon integrity and strength compared to the other groups (19).

However, it seems that ACR and RCA meet each other at some point, so there is no clarity of concepts. We found that RCA can be, in fact, ACR depending on the position where the LHBT is placed, acting as a mechanical augment for partial or complete repair.

#### Augmentation of subscapularis repair

##### Introduction

**Figure 4:**
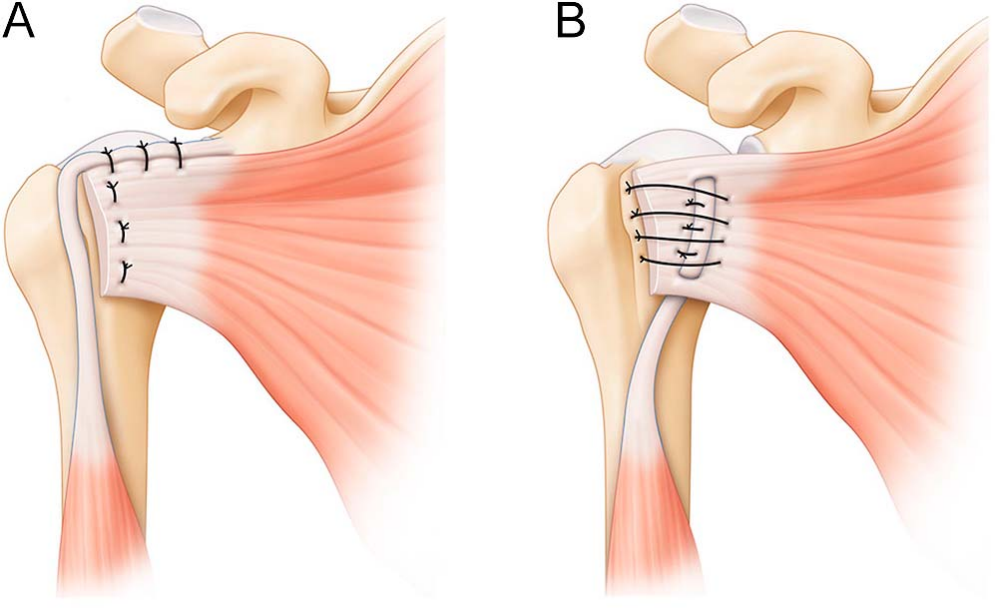
Illustrations of LHBT augmentation for subscapularis repair. In some cases, increasing the strength of the subscapularis tendon is useful. In these figures, some options are described: (A) the intra-articular portion of the LHBT is placed on the upper part of the SSc tendon at the rotator interval to reinforce the strength of the fixation, (B) the biceps-subscapularis sling technique requires that LHTB is passed through the subscapularis tendon vertically. (LHBT, long head of the biceps tendon, SSc, subscapularis). Reproduced with permission from https://beemed.com/.

Armstrong *et al.* found ultrasound evidence of subscapularis repair failure in 13% of patients with 8-month follow-up after total shoulder replacement. If patient dissatisfaction is significant, revision repair, tendon transfer or conversion to reverse arthroplasty may be considered (78) (Fig. 4).

##### Biomechanics

Hawthorne *et al.* found that using LHBT in subscapularis repair after shoulder arthroplasty resulted in significantly greater stiffness and loading failure compared to standard repair. This suggests that LHBT can improve the biomechanical strength of subscapularis repair (79).

##### Current techniques

Denard *et al.* (7) described a technique in which the LHBT is tenodesed on the upper border of the pectoralis major, harvested and then augmented to the native subscapularis tendon during shoulder arthroplasty as an autograft (Table 2) (7).

**Table 2:** Summary of research papers of subscapularis repair with LHBT techniques.

Study	Country	IND/CONTRA	Technique	Results	Conclusions
Martinho *et al.* (80)	Switzerland, Japan, France	IND: Large SSC tears; SSC repair possible only under tension; poor tissue quality	• SSC repair • LHBT tenotomy and tenodesis • LHBT transfer to rotator interval • SSC augmentation with use of LHBT as superior SSC cable	Biological and mechanical advantages at a lower cost without donor-site morbidity using LHBT	Alternative to improving the healing potential in SSC tears with an increased risk of failure. Further studies are needed to assess the biomechanical properties of this technique and the clinical performance
		CONTRA: Spontaneous rupture of LHBT or previous tenotomy or tenodesis			
Hawthorne *et al.* (79)	USA	IND: To avoid subscapularis failure following anatomic shoulder arthroplasty	• SSC repair in three groups • Control std repair • Horizontal biceps augmentation • V Shape biceps augmentation	No differences in cyclic displacement between the three groups. Horizontal and V-shape augmentation demonstrated significantly greater load to failure compared with traditional repair. V-shape repair had significantly greater load to failure compared to the horizontal repair. Horizontal and V-shape repairs demonstrated significantly greater stiffness compared to the traditional repair	Subscapularis peel repair augmentation with LHBT autograft following aTSA confers greater time zero load to failure and stiffness when compared to a standard subscapularis peel repair
Cohn *et al.* (81)	USA	IND: To avoid Subscapularis repair failure following aTSA; patients with an elevated risk of subscapularis failure following aTSA: age, smoking status, poor tissue quality, inappropriate physical therapy; oversized components; denervation injuries during subscapularis release	• LHBT is released from the supraglenoid tubercle and is passed through the sub- scapularis tendon vertically • Transosseous sutures are used to complete the peel repair, with the LHBT acting as a rip-stop to help protect the repair from suture pullout	LHBT offers an accessible, inexpensive, and autologous graft. The technique described is reproducible, adds minimal time to the total procedure, does not require changes in postoperative protocols and can be used with LTO, tenotomy, and peels	The use of the LHBT as a soft tissue augment has produced positive clinical results in other surgical settings, its use in subscapularis repair augmentation remains novel
Denard *et al.* (7)	USA	IND: Subscapularis repair following shoulder arthroplasty. REC: The only requirement for considering LHBT augmentation of SSc repair is the presence of the proximal attachment of LHBT. The presence of fraying or synovitis of the LHBT encountered intraoperatively does not necessarily exclude the patient for biological augmentation	• Tenodese the biceps tendon to the upper border of the pectoralis major tendon • Harvest the proximal segment of the long head biceps tendon and place in saline solution (0.9%) • Proceed with subscapularis takedown to access the glenohumeral joint and complete shoulder arthroplasty • Measure and cut a 27 mm long section of the biceps tendon • Compress the biceps tendon in trays for 4 min • Sew the compressed biceps graft into the native subscapularis tendon	Further studies are clearly required to investigate this technique. First, a biomechanical investigation would be valuable to determine if the augmentation improves load to failure or minimizes displacement. Most importantly, a clinical study evaluating healing would be valuable	Technique for compressing the biceps into a flat graft for autologous biological augmentation of subscapularis repair, following shoulder arthroplasty instead of discarding it

CONTRA, contraindications; IND, indications; SSC, subscapularis; LHBT, long head of the biceps tendon; aTSA, anatomic total shoulder arthroplasty; LTO, lesser tuberosity osteotomy; REC, recommendations.

Martinho *et al.* reported a technique using the LHBT to augment the subscapularis in its repair as an autograft, providing potential advantages at a lower cost and without donor site morbidity (80).

Cohn *et al.* (81) described the ‘biceps-subscap sling’ technique to repair subscapularis failure post-shoulder arthroplasty or in high-risk patients. The technique involves releasing the LHBT from the supraglenoid tubercle, passing it vertically through two openings in the subscapularis and completing the repair with transosseous sutures (81).

#### LHBT in the treatment of glenohumeral instability

##### Introduction

**Figure 5:**
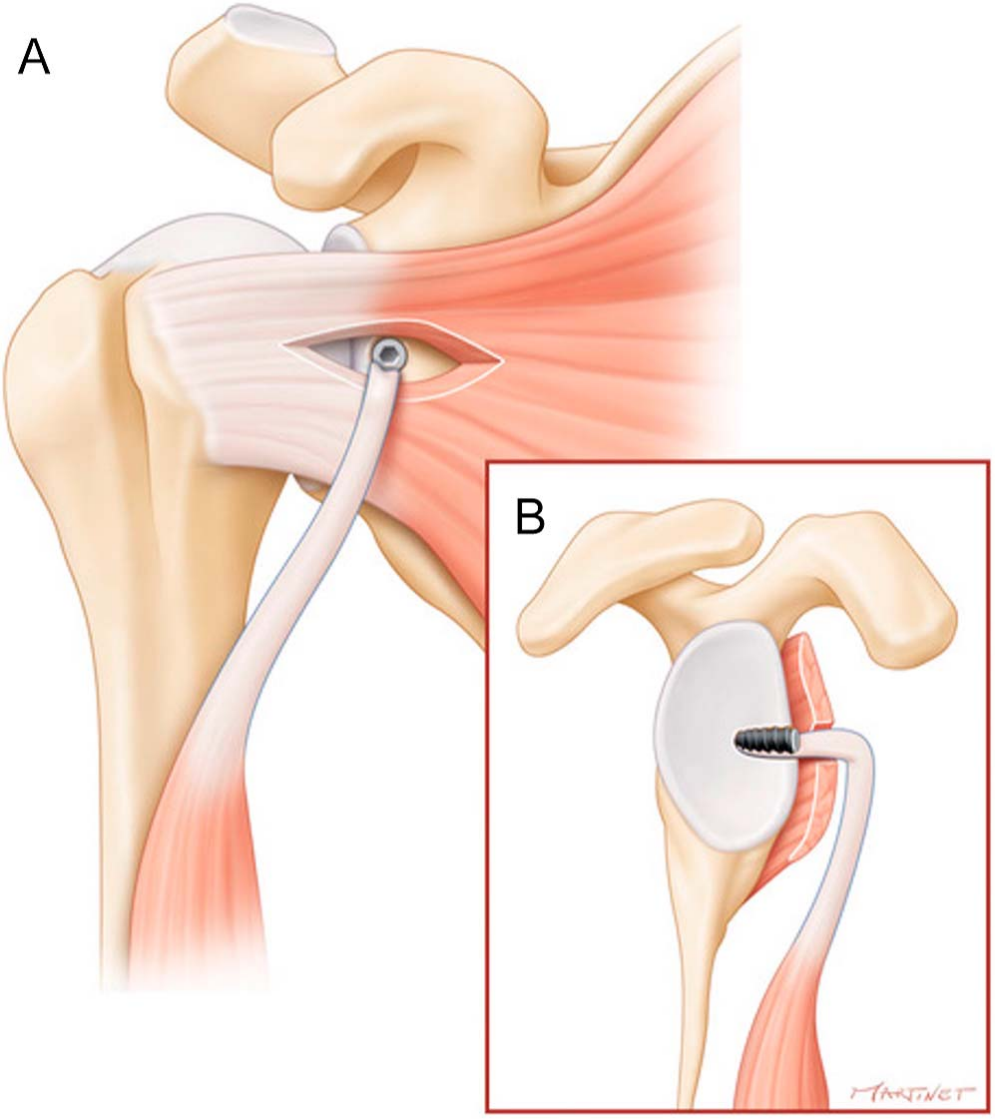
Image of the final construct of the shoulder's in-lay Dynamic anterior stabilization (DAS). (A) frontal view, (B) sagittal view. Reproduced with permission from https://beemed.com/.

The concept of DAS (see Supplementary Material 2) is a surgical technique used to treat anteroinferior glenohumeral instability by transferring the LHBT to the anterior glenoid margin to prevent translation of the humeral head (82), creating a ‘sling effect’ and hammock effect (83) (Fig. 5).

The lower limit of the anterior bone defect is 13.5% (84, 85, 86, 87), leaving a gray area of 13.5–20%, known as a subcritical bone defect, where defining the appropriate treatment has not been possible (88, 89). Biomechanical analyses suggest that DAS with Bankart repair can restore anterior glenohumeral stability, even in patients with up to 15% glenoid deficit (86, 90). The satisfactory results require a defect limit of 20% (83, 86, 91).

##### Biomechanical studies

Bokshan *et al.* (92) found that the LHBT provides better resistance to anterior translation during mid-range shoulder abduction compared to the conjoint tendon transfer (92). Mehl *et al.* compared Bankart standard repair with DAS. They found that both reduced anterior glenohumeral translation, with DAS showing less relative anterior translation, especially in cases of minor glenoid defects (85). Lobao *et al.* evaluated and compared LHBT transfer with Bankart repair and Latarjet, reporting that LHBT was a safe treatment option providing superior stabilization compared with Bankart repair, but inferior stabilization when compared with Latarjet in subcritical glenoid defects (93). Zacharias *et al.* (9) conducted a biomechanical cadaveric study proposing the use of LHBT to reconstruct the anterior glenoid labrum in the treatment of shoulder instability (3). They suggested tenotomy of the LHBT at the level of the pectoralis major and use of the proximal stump for labral reconstruction, resulting in significantly greater maximum force than the deficient and intact labrum (9).

##### Described techniques

Milenin and Toussaint described a DAS onlay procedure, fixing the LHBT parallel to the glenoid rim to create a neolabrum (94). Garcia *et al.* used an adjustable loop device for LHBT fixation, providing greater stability. (95) Zhao *et al.* developed a four-layer reconstruction technique for recurrent anterior shoulder dislocation, involving bone blocks, tunnel drilling, LHBT adjustment and capsule and labrum repair (96).

##### Clinical results

Collin *et al.* found that 91% of patients showed significant postsurgical improvement in Rowe score. ROM remained unchanged, and only three patients experienced recurrence with no reported complications (8). Kohan *et al.* studied LHBT suspensionplasty for multidirectional instability and found positive results after 3.2 years (97). De Campos Azevedo and Angelo treated three patients for chronic glenohumeral instability using arthroscopic onlay DAS (31, 98). All patients showed excellent results at 1-year follow-up, with one patient demonstrating complete range of motion and no complications over 16 months (99).

## Conclusion

The LHBT is a valuable source of living cells for shoulder surgeons. It is biomechanically strong, cost-effective and readily accessible. LHBT shows promise in addressing high failure rates associated with allografts for RCR and serves as an innovative option for subcritical bone loss in anterior shoulder instability. In addition, it enhances the resistance of the subscapularis in total shoulder arthroplasty. This narrative review summarizes the existing literature and its applications in shoulder surgery, emphasizing the need for high-level studies to validate the presented results.

- The LHBT is a biomechanically strong, cost-effective and accessible structure for its use in shoulder surgery.
- In the setting of rotator cuff, it is useful for supraspinatus irreparable rotator cuff tears as SCR, augmentation of repairable supraspinatus tendon tears both as augmentation and ACR.
- The LHBT has been described as augmentation of subscapularis repair in total shoulder replacement.
- Instability with subcritical bone loss is treated properly with the transfer of the LHBT to the glenoid rim in the DAS procedure, and biomechanical studies have shown its superiority to Bankart repair alone.

## Supplementary materials



## ICMJE Statement of Interest

Alexandre Lädermann is a paid consultant for Arthrex, Stryker, Medacta and Enovis. He received royalties from Stryker and Medacta. He is the (co-) founder of FORE, Med4Cast and BeeMed. He owns stock options in Medacta and Follow Health. He is on the board of the French Arthroscopic Society.

## Funding Statement

FORE (Foundation for Research and Teaching in Orthopedics, Sports Medicine, Trauma and Imaging in the Musculoskeletal System) Grant # FORE 2024-15.
