# Supplementary material for: Reinforcements and augmentations with the long head of the biceps tendon in shoulder surgery: a narrative review
Source: EFORT Open Rev. 2025 May 5;10(5):297–308. doi: 10.1530/EOR-2024-0122 (PMC12061019; doi:10.1530/EOR-2024-0122)
Supplement: Supplementary file 1 [file supplementary_materials.pdf]

SUPPLEMENTARY MATERIAL 1. Summary of research papers in SCR

AUTOR/ YEAR	COUNTRY	INDICATION/ CONTRAINDICATIONS	TECHNIQUE	RESULTS	CONCLUSIONS
**A. BOUTSIADIS(1)**<br>**2017**	China/<br>France	For an irreparable RCT.<br>The LHBT must be of good quality.<br>Do not perform when LHBT is excessively degenerated or partially torn.	SCR with autologous LHBT.<br>• Insert an anchor at supraspinatus footprint.<br>• Tenotomize distal part of LHBT and preserve its glenoid insertion.<br>• Fix the LHBT onto greater tuberosity.<br>• Suture side to side with infraspinatus and/or rotator interval.	SCR with LHBT protect the repaired tendons in favor of their healing process.	This technique transforms the LHBT into an efficient superior static stabilizer without any materials on glenoid.
**Y. KIM(2)**<br>**2018**	Korea	For an irreparable RCT.<br>The LHBT must be of good quality.<br>Do not perform when LHBT is excessively degenerated or partially torn.	SCR in situ by re-routing LHBT.<br>• Debridement soft tissue around LHBT.<br>• Decortication of footprint for rerouting the LHBT.<br>• Lateral and medial insertion of anchor to fix the LHBT.<br>• Insert another anchor just posterior to the lateral LHBT anchor.<br>• Repair rotator cuff with sutures from 3 anchors.	This technique re-routing the LHBT creating a downward force to humeral head.<br>This technique is reliable, simple, and without morbidity risk.	This is a reasonable option for younger patients with posterosuperior large/massive RCTs to avoid tendon transfer or reverse total shoulder arthroplasty.

AUTOR/ YEAR	COUNTRY	INDICATION/ CONTRAINDICATIONS	TECHNIQUE	RESULTS	CONCLUSIONS
**K. HERMANOWICZ (3) 2018**	Poland	For massive irreparable supraspinatus and infraspinatus tears with muscle degeneration and fatty infiltration exceeding 50%, intact or reparable subscapularis.<br>Do not perform in arthrosis glenohumeral, LHBT damaged closer than 4 cm from the labral footprint or SLAP III-IV grade.	SCR the "biceps patch" technique<br>• Bone bed preparation of great tuberosity<br>• The "lasso-loop" technique is applied to fix LHBT.<br>• Suture is introduced through the infraspinatus remnant from the subacromial to the articular side.<br>• Another suture is passed through the supraspinatus and LHBT from the subacromial to the articular side.<br>• Fist suture is passed through the LHBT and supraspinatus on its subacromial side.<br>• Second suture pass through the infraspinatus, LHBT, and supraspinatus.<br>• This suturing technique creates a "sandwich" system, where the LHBT is positioned between 2 rotator muscles.	This "biceps patch" technique fix the LHBT on greater tubercle and leave the proximal footprint intact, the superior capsule was reconstructed in the most natural way.<br>The applied mattress suture provides more strength for whole complex.	The "biceps patch" allows to restore full active ROM, LHBT is natural graft, small number of implants, minimized costs, not technically demanding and is reproducible.<br>But it could cause Shoulder pain up to 6 mo. after surgery.
**C. CHILLEMI(4) 2018**	Italy	Irreparable posterior/superior RCT.<br>Without glenohumeral arthritis and stiffness.<br>The LHBT must be of good quality.<br>Do not perform when LHBT is excessively degenerated or partially torn.	SCR of the shoulder: the ABC technique.<br>• Decortication of the footprint.<br>• Tenotomize distal part of LHBT maintaining intact its glenoid origin.<br>• Two- anchor or two- trans osseous lateral tunnels to fix LHBT.<br>• Perform partial side-to-side repair passing through the infraspinatus tendon.	This technique did in 9 patients is safe without an increase postoperative pain in the first 6 months.	This technique allows an all-arthroscopic SCR in a safe, easier, time and cost-saving way.

AUTOR/ YEAR	COUNTRY	INDICATION/ CONTRAINDICATIONS	TECHNIQUE	RESULTS	CONCLUSIONS
**D. KIM(5)**<br>**2019**	Korea	For irreparable massive RCT.<br>“The snake technique”<br>Good quality of the LHBT anchors to the glenoid labrum (normal or <20% partial tear).<br>Minimal or no glenohumeral arthritis.	SCR with autologous LHBT.<br>• Humeral and glenoid bone bed preparation and anchors insertion.<br>• Biceps tenodesis and Biceps autograft preparation.<br>• Partial repair.	This technique can modify the size of reconstructed capsule by adjusting the number of bundles in the operation.<br>Better proprioception and healing by maintained blood supply.	This technique would be a useful surgical treatment option for irreparable massive RCT.<br>The biceps tendon stretches and increases its tension might cause pain at early stage after surgery.
**C. CHIANG(6)**<br>**2019**	Taiwan	For repairable o irreparable large to massive RCTs.<br>The LHBT must be of good quality.<br>Do not perform when LHBT is excessively degenerated or partially tear more than 30%.	SCR with LHBT autograft.<br>• Insert suture anchor to fix the LHBT in the medial and medial part of the footprint.<br>• Fix the LHBT by passing a suture through its anterior and posterior third.<br>• Tenotomy of the LHBT.<br>• Repair of the RCT by single-row or suture bridging technique. The LHBT was sandwiched between the repaired rotator cuff and the footprint.	This technique can be easily transposed from intra-articular space and does not require extra incisions.<br>Besides the blood supply from the bone bed of the footprint, the transposed LHBT relieves the upward tension and provides extra supplies of nutrient and blood for healing of RCT.	This technique could offer promising clinical results, with low cost.<br>The disadvantages could be related to LHBT tenotomy such as reduced strength in flexion and supination of the elbow, Popeye's sign deformity and painful biceps cramps
**E. FANDRIDIS(7)**<br>**2020**	Greece	SCR with massive irreparable RCT. “Box configuration”.<br>• LHBT integrity or tear <30%, SLAP < III grade.<br>• Intact teres minor without or minimal (stage 1) fatty infiltration.<br>Do not perform in glenohumeral OA Hamada >3, subscapularis tendon tear Lafosse V, with anterosuperior scape, deltoid muscle atrophy or axillary nerve injury, with active elevation at scapular plane <45° with normal passive ROM.	SCR with double-bundle LHBT autograft.<br>• Decortication of the footprint.<br>• Tenotomize LHBT 1cm above musculotendinous junction and secure proximal part.<br>• Fix with suture-anchor/button the musculotendinous part of LHBT.<br>• Insert one or two anchors at 10-11 o’clock labrum positions, another two in great tuberosity.<br>• Suture infraspinatus with posterior bundle of LHBT.<br>Final construct as “ABCD”.	Preserves LHBT vascularity keeping its origin at labrum, avoids complications such as Popeye’s sign, loss of supination and pain during resisted flexion.	Double-bundle technique of LHBT may imitate superior capsule more anatomically than the single-bundle techniques.

AUTOR/ YEAR	COUNTRY	INDICATION/ CONTRAINDICATIONS	TECHNIQUE	RESULTS	CONCLUSIONS
**ADRIAN S.(8)** **2020**	USA	Massive rotator cuff tears<br><br>The technique cannot be used if the LHBT is ruptured or very severely damaged.	Bio-SCR technique<br>• Prepare bone bed at greater tuberosity.<br>• Release transverse humeral lig.<br>• Mobilize and fix LHBT into greater tuberosity.<br>Repair RC.	This technique provides a static, and dynamic downforce that could maintain glenohumeral congruity.<br>incorporation of LHBT into RCR adds native tissue, rich in tenocytes and fibroblasts.	Technically easy and inexpensive alternative.<br>The effects related to local biceps symptoms or function have not been fully elucidated.
**G. MILANO(9)** **2020**	Italy	For large-massive repairable posterosuperior cuff tears delaminated with severe retraction and poor mobility that are still repairable.<br>Do not perform in case of irreparable tears, involvement of the teres minor, cuff tear arthropathy, and shoulder stiffness.	The Arthroscopic "Cuff-Plus" Technique<br>• Microfractures of greater tuberosity<br>• Suture through LHBT with a "lasso-loop" configuration.<br>• Tenotomize the LHBT distally to the sutures.<br>• Reroute posteriorly proximal stump of LHBT to fix with anchor onto supraspinatus footprint.<br>• Place anchors anterior and/or posterior to LHBT along articular margin, depending on tear configuration.<br>Knot the sutures from posterior to anterior according to the direction of the vectors	LHBT may improve cuff healing by a tension-free repair and biological augmentation which could decrease rotator cuff retear rate, especially in large-massive tears.	Possible lower control of superior escape of humeral head for small surface coverage rather vs standard SCR.<br>Proximal stump of LHBT is a potential source of postoperative pain.
**D. KIM(10)** **2020**	Korea	For partially repairable massive RCT.<br>LHBT is normal or damaged < 20%.	Arthroscopic partial SCR with LHBT: L-shape shifting technique.<br>• Prepare bone bed in greater tuberosity.<br>• Insert an anchor where posterior RC and LHBT would overlap and be sutured.<br>• Suture through and wraps LHBT at anchor site.<br>• Anterior and posterior RCR.	LHBT gives downward forces to the humeral head and serves as interposition between RCT and humeral head.	This technique can be used in massive RCTs, which are not completely repairable.<br>Biceps interposition makes superior capsule thicker and harder, enhance biological healing and strengthening poor quality rotator cuff.

AUTOR/ YEAR	COUNTRY	INDICATION/ CONTRAINDICATIONS	TECHNIQUE	RESULTS	CONCLUSIONS
**B. TERRA(11)** **2021**	Brazil	For massive irreparable RCTs<br>The LHBT must be of good quality.<br>Do not perform when LHBT is excessively degenerated or partially tear more than 30%.	SCR with LHBT autograft.<br>• Prepare bone bed for LHBT at great tuberosity.<br>• Insert one anchor at center, one at posterosuperior and one just lateral to humeral head cartilage.<br>• Mobilize LHBT to humeral head center.<br>• Incorporate biceps to reinforces RCR<br>• Biceps tenotomy is not required.<br>•	No tenotomized LHBT gives downward forces to humeral head and increase acromiohumeral distance.<br>Overlap damaged RC with LHBT makes SCR thicker, harder and enhance biological healing.	SCR can perform only by posteriorizing and fixing the LHBT onto the greater tuberosity at center of humeral head.
**LLANOS RODRIGUEZ(12)** **2021**	Spain	For large to massive RCT.<br>The LHBT must be of good quality.<br>Do not perform when LHBT is excessively degenerated or partially torn.	Anterior capsular reconstruction with proximal biceps tendon.<br>• Debridement soft tissue around the LHBT<br>• Create a trough for rerouting the LHBT.<br>• Anchor insertion in trough to fix de LHBT.<br>• Transfer and tenodesis of LHBT.<br>• RCR	This technique restricts superior migration of humeral head, relieving tension over repaired RCT.	Effective modification of an SCR using the LHBT to treat massive RCT.
**BRANDÃO BL(13)** **2021**	Brazil	For young and active patients with irreparable RCT.<br>It is important length and integrity of LHBT for the graft.	SCR with LHBT: The Biceps Loop Technique<br>• Tenodesis of LHBT at inferior border of pectoralis major.<br>• Tenotomize immediately proximal to tenodesis and suture with Krakow stich.<br>• Drill a bone tunnel at great tuberosity. Pass ends of Krackow suture into a loop and pull.<br>• Fix posterior bundle of LHBT at posterior glenoid rim.<br>• Partial repair of RC to medial aspect of footprint.	Transfer LHBT into a bone tunnel create larger contact area to improve healing and reduces suture anchors.<br>Tenotomy with tenodesis may reduce pain while preserve biceps strength and cosmesis.	Use of intra and extra-articular portion of LHBT could recreate joint fulcrum avoiding progression to cuff tear arthropathy.<br>Pain is a possible for over tensioned LHBT and humeral head fracture during tunnel drilling.

AUTOR/ YEAR	COUNTRY	INDICATION/ CONTRAINDICATIONS	TECHNIQUE	RESULTS	CONCLUSIONS
**BHATIA DN(14) 2021**	India	For MCT	MCT: The Biceps-Cuff Bursa Composite Repair.<br>• Arthroscopic evaluation is performed to identify the rotator cuff tear patter.<br>• Subescapularis repair biceps<br>• Take autograft LHBT prior to tenodesis.<br>• Dissect carefully subacromial bursa from the posterior and posterolateral deltoid fascia.<br>• Preserve medial and lateral bursa and vascular sheet along the tendon aspect.<br>• Release torn and retracted tendons.<br>Debride greater tuberosity and repair rotator cuff with three or four suture anchors in a single-row configuration.	"sandwiched" degenerative rotator cuff between both autografts provide superior restraint and potential regenerative for repair a chronic massive tear.<br><br>There is no need for additional patches or biological scaffolds.	Subacromial bursal cells has the possible advantages of increased healing and better incorporation of the cuff at the tendon-bone.<br><br>.The subacromial bursa extends from medial to lateral across the posterosuperior rotator cuff and is relatively thicker and structurally stronger medially and posteriorly<br><br>During bursal augmentation, medial and lateral bursal attachments should be kept intact for preserving vascularity, and the entire sheet should be mobilized as a single structure and sutured to the tendon during cuff repair.9 A possible disadvantage with this technique may be the inability to predict the thickness and ability to dissect an intact vascular bursal layer

AUTOR/ YEAR	COUNTRY	INDICATION/ CONTRAINDICATIONS	TECHNIQUE	RESULTS	CONCLUSIONS
**DENARD P(15)** **2021**	USA	May have a role in augmentation of an irreparable massive cuff tear	in situ biceps tenodesis and a box-shaped LHBT SCR in a superior massive MCT model	ROM was not impaired with either repair construct (P > .05). The box SCR decreased superior translation by approximately 2 mm compared with the MCT at 0°, but translation remained greater compared with the intact state in nearly every testing position. The in situ tenodesis had no effect on superior translation. Peak subacromial contact pressure was increased in the MCT at 0° and 20° abduction compared with the native state but not different between the native and box SCR at the same positions	This technique partially restores increased superior translation and peak subacromial contact pressure due to MCT. The technique may have a role in augmentation of an irreparable MCT
**LIN J(16)** **2019**	China	Repairing a full-thickness RC tear<br>LHBT lesions and RC tears	- initial assessment and acromioplasty<br>- transposition of the long head of biceps<br>- fixation of the long head of biceps<br>- transection of the LHBT proximal to the knot of the suture near the bicipital groove<br>- stabilization the long head of biceps<br>- rotator cuff repair<br>transection of the LHBT in the supraglenoid tubercle	No intra-operative complication no infection occurred.<br>At 6-month post operation VAS score decreased and Constant score increased.	Transposition of the long head of biceps is a choice for full-thickness RC tear. Further, we need more clinical and biomechanical research to support this novel technique.

RC: Rotator Cuff, RCR: rotator cuff repair, MCT: Massive Cuff Tear, SCR: Superior Capsular Reconstruction, LHBT: Long Head of the Biceps Tendon

REFERENCES

1. Boutsiadis A, Chen S, Jiang C, Lenoir H, Delsol P, Barth J. Long Head of the Biceps as a Suitable Available Local Tissue Autograft for Superior Capsular Reconstruction: “The Chinese Way”. Arthroscopy Techniques. 2017;6(5):e1559-e66.
2. Kim Y-S, Lee H-J, Park I, Sung GY, Kim D-J, Kim J-H. Arthroscopic In Situ Superior Capsular Reconstruction Using the Long Head of the Biceps Tendon. Arthroscopy Techniques. 2018;7(2):e97-e103.
3. Hermanowicz K, Góralczyk A, Malinowski K, Jancewicz P, Domżalski ME. Long Head Biceps Tendon—Natural Patch for Massive Irreparable Rotator Cuff Tears. Arthroscopy Techniques. 2018;7(5):e473-e8.
4. Chillemi C, Mantovani M, Gigante A. Superior capsular reconstruction of the shoulder: the ABC (Arthroscopic Biceps Chillemi) technique. Eur J Orthop Surg Traumatol. 2018;28(6):1215-23.
5. Kim D, Jang Y, Park J, On M. Arthroscopic Superior Capsular Reconstruction With Biceps Autograft: Snake Technique. Arthroscopy Techniques. 2019;8(10):e1085-e92.
6. Chiang CH, Shaw L, Chih WH, Yeh ML, Su WR. Arthroscopic Rotator Cuff Repair Combined With Modified Superior Capsule Reconstruction as Reinforcement by the Long Head of the Biceps. Arthroscopy Techniques. 2019;8(10):e1223-e31
7. Fandridis E, Zampeli F. Superior Capsular Reconstruction With Double Bundle of Long Head Biceps Tendon Autograft: The “Box” Technique. Arthroscopy Techniques. 2020;9(11):e1747-e57.
8. Adrian SC, Field LD. Biceps Transposition for Biological Superior Capsular Reconstruction. Arthroscopy Techniques. 2020;9(6):e841-e6.

9. Milano G, Marchi G, Bertoni G, Vaisitti N, Galli S, Scaini A, Saccomanno M. Augmented Repair of Large to Massive Delaminated Rotator Cuff Tears With Autologous Long Head of the Biceps Tendon Graft: The Arthroscopic "Cuff-Plus" Technique. Arthroscopy Techniques. 2020;9(11):e1683-e8.
10. Kim D-S, Yeom J, Park J, Cha J. L-Shape Superior Capsular Augmentation Technique Using Biceps Tendon: The Biceps L-Shape Shifting Technique. Arthroscopy Techniques. 2020;9(6):e703-e9.
11. Terra BB, Sassine TJ, Ejnisman B, de Castro Pochini A, Belangero PS. Arthroscopic partial Superior Capsular Reconstruction using the Long Head of the Biceps Tendon–Technique Description. Arthroscopy Techniques. 2021;10(3):e669-e73.
12. Llanos-Rodríguez Á, Escandón-Almazán P, Espejo-Reina A, Nogales-Zafra J, Egozgue-Folgueras R, Espejo-Baena A. Anterior Capsular Reconstruction With Proximal Biceps Tendon for Large to Massive Rotator Cuff Tears. Arthroscopy Techniques. 2021;10(8):e1965-e71.
13. Brandão BL, Soares da Fonseca R, Zaluski AD, Gribel Carneiro B, Cohen MT, da Rocha Motta Filho G. Superior Capsular Reconstruction using the Long Head of the Biceps Tendon: The Biceps Loop Technique. Arthroscopy Techniques. 2021;10(6):e1647-e53.
14. Bhatia DN. Arthroscopic Biological Augmentation for Massive Rotator Cuff Tears: The Biceps-Cuff-Bursa Composite Repair. Arthroscopy Techniques. 2021;10(10):e2279-e85.
15. Denard PJ, Chae S, Chalmers C, Choi JH, McGarry MH, Adamson G, Park M, Lee TQ. Biceps Box Configuration for Superior Capsule Reconstruction of the Glenohumeral Joint Decreases Superior Translation but Not to Native Levels in a Biomechanical Study. Arthrosc Sports Med Rehabil. 2021;3(2):e343-e50.

16. Lin J, Qi W, Liu Z, Chen K, Li X, Yan Y, Xu X, Xue X, Yang Y, Pan X. An arthroscopic technique for full-thickness rotator cuff repair by transposition of the long head of biceps. Orthop Traumatol Surg Res. 2019;105(2):265-9.

SUPPLEMENTARY MATERIAL 2. Summary of research papers for DAS techniques

AUTOR/ YEAR	COUNTRY	INDICATION/ CONTRAINDICATIONS	TECHNIQUE	RESULTS	CONCLUSIONS
**COLLIN / LÄDERMANN(1)**<br>**2018**	France<br>Switzerland	For anteroinferior glenohumeral instability<br>Limited bone defects associated with SLAP<br>Overhead athletes and thrower	Dynamic Anterior Stabilization Using the Long Head of the Biceps for Anteroinferior Glenohumeral Instability<br>• Portal placements<br>• Anterior portals under endoscopic control<br>• Bicipital groove is opened laterally and distally to avoid detaching the subscapularis.<br>• Use a switching stick through a posterior approach to retract the subscapularis during the split		It is believed that DAS presented here will provide a promising alternative to the surgical treatments for anteroinferior glenohumeral instability
**MEHL J(2)**<br>**2019**	USA	Dynamic Anterior Shoulder Stabilization With LHBT	Anterior shoulder instability with 10% - 20% of glenoid bone defect.<br><br>Compare dynamic anterior shoulder stabilization alone vs Bankart repair with dynamic anterior shoulder stabilization	DAS shows better stability by moving humeral head posteriorly and enhance the relative anterior glenohumeral translation.	DAS technique provided decreased anterior glenohumeral translation in cases of Bankart lesions with limited anterior bone loss of 20%.

AUTOR/ YEAR	COUNTRY	INDICATION/ CONTRAINDICATIONS	TECHNIQUE	RESULTS	CONCLUSIONS
**KANG(3)** **2021**	China	Anterior Shoulder instability with 20% glenoid defect	Bankart repair with transferred cojoined tendon LHBT with 20% anterior shoulder instability. • Bankart repair was performed with 3 suture anchors at the 3:30-, 4:30-, and 5:30-o'clock. • A 2.5-mm glenoid tunnel was established, located on the midportion of the defect and 7 mm medial to the defect rim. • Suture of the conjoined tendon or LHBT was passed through the tunnel and fixed by miniplate on the back of glenoid neck.	This technique re-routing the LHBT creating a downward force to humeral head. This technique is reliable, simple, and without morbidity risk.	This is a reasonable option for younger patients with posterosuperior large/massive RCTs to avoid tendon transfer or reverse total shoulder arthroplasty.
**LOBAO M(4)** **2022**	USA	Chronic anterior shoulder instability with and without subcritical bone loss • For patients with high recurrence risk • Subcritical glenoid bone loss or poor capsule labral tissue quality. • Military's, throwers, and high-performance athletes.	Transfer LHBT to augment Bankart repair.	DAS techniques, as augmentation, improve dislocation resistance to the same resistance as the contralateral arm.	These findings suggest that LHBT may be effective in the presence of a 20% subcritical anterior glenoid bone defect.
**NICHOLSON A.(5)** **2022**	USA	Dynamic anterior stabilization with Bankart repair<br><br>The propose was to evaluate the biomechanical effects of the DAS technique in shoulders with 15% glenoid bone loss in cadaveric specimens and to examine DAS with Bankart	DAS with Bankart repair was performed similarly to the technique described by Collin and Lädermann	Creation of a trans subscapularis biceps sling restores anterior stability to a similar degree as an isolated Bankart repair or glenoid with 15% bone loss.	Dynamic anterior stabilization with Bankart repair offers the potential for even more anterior restraint even in a bone loss defect around 15%
**ZACHARIAS(6)** **2023**	USA	Anterior shoulder instability repair<br><br>Biomechanical cadaveric study	LHBT tenotomy at pectoralis major and use of proximal stump as graft for anterior	Reconstructed labrum had significantly	Long-term clinical data will be needed to confirm the benefit of

AUTOR/ YEAR	COUNTRY	INDICATION/ CONTRAINDICATIONS	TECHNIQUE	RESULTS	CONCLUSIONS
			labrum reconstruction	greater peak force than the deficient labrum and intact labrum	this autologous labral reconstruction in vivo
**MILENIN & TOUSSAINT(7)** **2019**	Russia France	Chronic traumatic anteroinferior instability without substantial bone loss and weakness of the glenohumeral ligaments and labrum Technique that provides triple mechanisms of stabilization like Latarjet	DAS with simultaneous plasty of the anterior segment of the labrum with LHBT fixed parallel to the glenoid rim creating a neolabrum and anterior bumper effect as a soft-tissue block	By creating triple mechanisms of stability (bumper effect, reinforcement of ligaments, and sling effect), this procedure can significantly reinforce the Bankart procedure in cases of poor-quality glenohumeral ligaments without significant bone loss (<20%)	Future research is necessary to study incidents of recurrence, bicep pain, range of motion restrictions, and the possibility of using this technique for professional athletes
**CAMPOS AZEVEDO(8)** **2021**	Portugal	Chronic traumatic GH anteroinferior instability without substantial bone damage of the glenoid and humeral head, with poor quality of GH ligaments and labrum	DAS with LHBT fixed on the anteroinferior glenoid using a novel double double-pulley all-suture anchor method	Excellent 12-month clinical and imaging outcomes, with substantial improvements in WOSI and Rowe score	Modification of DAS described in this case report may be a safe and reliable treatment option for chronic anteroinferior glenohumeral instability with Bankart and Hill-Sachs lesions with limited (<13.5%) to subcritical (‡13.5%) GBL, and types I-III SLAP lesions but may be contraindicated in patients with critical (‡25%) GBL or LHB

AUTOR/ YEAR	COUNTRY	INDICATION/ CONTRAINDICATIONS	TECHNIQUE	RESULTS	CONCLUSIONS
					tears
**CAMPOS AZEVEDO(9)** **2023**	Portugal	Indications: AGHI and GBL ≤ 20% AGHI and anterior capsulolabral insufficiency, AGHI and SLAP type I-III Contraindications: AGHI and GBL>20%, MDI, Absent LHB, AGHI and SLAP type IV, Beighton score >6	DAS with onlay LHBT fix on the anterior glenoid rim	Better for patients in whom it is impossible to augment DAS with the Bankart repair because they have anterior capsulolabral insufficiency secondary to multiple dislocation episodes, the additional labroplasty effect of onlay DAS may provide an advantage over inlay DAS.	onlay DAS avoids some of the risks of complications of inlay DAS, either related to tunnel fracture, or to the presence of metallic hardware in the glenohumeral joint
**GARCIA JC(10)** **2022**	Brazil	Anterior shoulder instability with or without critical bone loss (13.5%)	DAS with adjustable loop device	This procedure is not substitute for bone block procedures when more than 20% of glenoid bone loss is present; instead, it will add more stability to the current soft-tissue procedures in the presence of smaller amounts of bone loss.	this surgical procedure and similar procedures can fit exactly in the gray zone between the Bankart and Bristow-Latarjet procedures. It could also be useful in athletes and high-demand patients

AUTOR/ YEAR	COUNTRY	INDICATION/ CONTRAINDICATIONS	TECHNIQUE	RESULTS	CONCLUSIONS
**ZHAO J(11) 2020**	China	Indications for 4-layer structural reconstruction<br>• Requirement of both soft tissue sling augmentation and bone fragment augmentation<br>Detailed indications for sling augmentation with the long head of the biceps<br>• Age <45 y<br>• Participation in competitive sports<br>• Requirement of forceful external rotation and abduction of the shoulder<br>• Capsule ligament deficiency<br>• Combined SLAP lesion<br>Contraindications for sling augmentation with the long head of the biceps<br>• Sling augmentation not required<br>• LHB cannot be used<br>Detailed indications for the bone fragment augmentation at the anterior glenoid<br>• Glenoid defect<br>• Requirement of osseous stimulation for labrum-glenoid remolding<br>Contraindications for the bone fragment augmentation at the anterior glenoid<br>• Glenoid defect not existing and osseous stimulation not required	2 bone blocks with biceps transfer with adjustable loop.<br>anchor.	Routine technique performed in our clinical practice, considering that most of our young and active patients need sling augmentation, that every patient needs osseous stimulation by glenoid bone grafting for better glenoid remodeling, and that capsule-labrum repair is critical to ensure capsule-labrum connection to the glenoid and seal the glenohumeral joint.<br>This technique is somewhat time-consuming: 90 to 120 minutes	They believe that this technique will enhance the field of anterior shoulder reconstruction for complicated anterior shoulder dislocation.

DAS: Dynamic Anterior shoulder Stabilization; AGHI: Anterior glenohumeral instability; MDI: multidirectional glenohumeral instability; SLAP: superior labrum anterior to posterior, RCT: Rotator Cuff Tear

REFERENCES

1. Collin P, Lädermann A. Dynamic Anterior Stabilization Using the Long Head of the Biceps for Anteroinferior Glenohumeral Instability. Arthroscopy Techniques. 2017;7(1):e39-e44.
2. Mehl J, Otto A, Imhoff FB, Murphy M, Dyrna F, Obopilwe E, Cote M, Lädermann A, Collin P, Beitzel K, Mazzoca A. Dynamic Anterior Shoulder Stabilization With the Long Head of the Biceps Tendon: A Biomechanical Study. Am J Sports Med. 2019;47(6):1441-50.
3. Kang Y, Wang L, Wang M, Wei Y, Li Y, Jiang J, Yu S, Zhao J, Xie G. Bankart Repair With Transferred Long Head of the Biceps Provides Better Biomechanical Effect Than Conjoined Tendon Transfer in Anterior Shoulder Instability With 20% Glenoid Defect. Arthroscopy. 2022;38(9):2628-35.
4. Lobao MH, Abbasi P, Murthi AM. Long head of biceps transfer to augment Bankart repair in chronic anterior shoulder instability with and without subcritical bone loss: a biomechanical study. J Shoulder Elbow Surg. 2022;31(5):1062-72.
5. Nicholson AD, Carey EG, Mathew JI, Pinnamaneni S, Jahandar A, Kontaxis A, Dines DM, Dines JS, Blaine TA, Fu MC, Rodeo SA, Warren RF, Gullota LV, Taylor SA. Biomechanical analysis of anterior stability after 15% glenoid bone loss: comparison of Bankart repair, dynamic anterior stabilization, dynamic anterior stabilization with Bankart repair, and Latarjet. J Shoulder Elbow Surg. 2022;31(11):2358-65.
6. Zacharias AJ, Platt BN, Rutherford M, Kamineni S. Shoulder Anteroinferior Glenoid Labrum Reconstruction With the Long Head of the Biceps Tendon Restores Glenohumeral Stability: A Cadaveric Biomechanical Study. Arthroscopy. 2023;39(2):196-201.
7. Milenin O, Toussaint B. Labral Repair Augmentation by Labroplasty and Simultaneous Trans-Subscapular Transposition of the Long Head of the Biceps. Arthroscopy Techniques. 2019;8(5):e507-e12

8. de Campos Azevedo C, Ângelo AC. All-Suture Anchor Dynamic Anterior Stabilization Produced Successful Healing of the Biceps Tendon: A Report of 3 Cases. JBJS Case Connect. 2021;11(1).
9. de Campos Azevedo CI, Ângelo AC. Dynamic Anterior Stabilization of the Shoulder: Onlay Biceps Transfer to the Anterior Glenoid Using the Double Double-Pulley Technique. Arthroscopy Techniques. 2023;12(7):e1097-e106.
10. Garcia JC, Mendes RB, Muzy PC, de Paiva Raffaelli M, Dumans e Mello MB. Dynamic Anterior Stabilization of the Shoulder With Adjustable-Loop Device. Arthroscopy Techniques. 2022;12(1):e39-e44.
11. Zhao J, Tang J. Four-Layer Structural Reconstruction for Recurrent Anterior Shoulder Dislocation. Arthroscopy Techniques. 2020;9(12):e2031-e40
